# Linking Electronic Health Record Prescribing Data and Pharmacy Dispensing Records to Identify Patient-Level Factors Associated With Psychotropic Medication Receipt: Retrospective Study

**DOI:** 10.2196/63740

**Published:** 2025-03-04

**Authors:** Peng Wu, Jillian H Hurst, Alexis French, Michael Chrestensen, Benjamin A Goldstein

**Affiliations:** 1 Department of Biostatistics and Bioinformatics, School of Medicine, Duke University, 2424 Erwin Road Suite 902, 9023 Hock Plaza, Durham, NC, United States, 1 919 681 5011; 2 Department of Pediatrics, School of Medicine, Duke University, Durham, NC, United States; 3 Department of Psychiatry and Behavioral Sciences, School of Medicine, Duke University, Durham, NC, United States; 4 Duke Health Technology Solution, Duke University Health System, Durham, NC, United States

**Keywords:** electronic health records, pharmacy dispensing, psychotropic medications, prescriptions, predictive modeling

## Abstract

**Background:**

Pharmacoepidemiology studies using electronic health record (EHR) data typically rely on medication prescriptions to determine which patients have received a medication. However, such data do not affirmatively indicate whether these prescriptions have been filled. External dispensing databases can bridge this information gap; however, few established methods exist for linking EHR data and pharmacy dispensing records.

**Objective:**

We described a process for linking EHR prescribing data with pharmacy dispensing records from Surescripts. As a use case, we considered the prescriptions and resulting fills for psychotropic medications among pediatric patients. We evaluated how dispensing information affects identifying patients receiving prescribed medications and assessing the association between filling prescriptions and subsequent health behaviors.

**Methods:**

This retrospective study identified all new psychotropic prescriptions to patients younger than 18 years of age at Duke University Health System in 2021. We linked dispensing to prescribing data using proximate dates and matching codes between RxNorm concept unique identifiers and National Drug Codes. We described demographic, clinical, and service use characteristics to assess differences between patients who did versus did not fill prescriptions. We fit a least absolute shrinkage and selection operator (LASSO) regression model to evaluate the predictability of a fill. We then fit time-to-event models to assess the association between whether a patient filled a prescription and a future provider visit.

**Results:**

We identified 1254 pediatric patients with a new psychotropic prescription. In total, 976 (77.8%) patients filled their prescriptions within 30 days of their prescribing encounters. Thus, we set 30 days as a cut point for defining a valid prescription fill. Patients who filled prescriptions differed from those who did not in several key factors. Those who did not fill had slightly higher BMIs, lived in more disadvantaged neighborhoods, were more likely to have public insurance or self-pay, and included a higher proportion of male patients. Patients with prior well-child visits or prescriptions from primary care providers were more likely to fill. Additionally, patients with anxiety diagnoses and those prescribed selective serotonin reuptake inhibitors were more likely to fill prescriptions. The LASSO model achieved an area under the receiver operator characteristic curve of 0.816. The time to the follow-up visit with the same provider was censored at 90 days after the initial encounter. Patients who filled prescriptions showed higher levels of follow-up visits. The marginal hazard ratio of a follow-up visit with the same provider was 1.673 (95% CI 1.463‐1.913) for patients who filled their prescriptions. Using the LASSO model as a propensity-based weight, we calculated the weighted hazard ratio as 1.447 (95% CI 1.257‐1.665).

**Conclusions:**

Systematic differences existed between patients who did versus did not fill prescriptions. Incorporating external dispensing databases into EHR-based studies informs medication receipt and associated health outcomes.

## Introduction

Electronic health record (EHR) data have become a vital data resource for health outcomes and health services research [1]. These data provide detailed patient information, including demographics, diagnoses, prescriptions, and procedures, which can facilitate comprehensive studies that reflect real-world clinical practice [2]. EHR data are increasingly being used to support clinical decision-making with prediction models [3,4] and assess population health [5]. Additionally, EHR data facilitate clinical trial simulation by assessing assumptions and guiding study design of the actual trials [6,7]. They have also emerged as a key resource for the observation of long-term drug safety and effectiveness [8,9], providing insights into drug benefits, interactions, and side effects [10]. Beyond this, EHR data provide valuable opportunities for uncovering disease phenotypes, mapping treatment pathways, and informing drug repurposing and safety studies [11,12]. The use of EHR data in pharmacoepidemiology research can further our understanding of drug impacts and access, driving advancements in care [13].

Despite the utility of EHR data, a significant limitation arises for research focused on medication use. While EHRs contain granular information on medication prescriptions, commercial pharmacy data are typically held in a separate database that must be connected to the EHR. Because these databases are separate, prescription fill data may not be readily available and are often not directly connected to a specific encounter [14,15]. This discrepancy can lead to inaccuracies in understanding which patients have actually received medication and how medication receipt may influence patient outcomes [16]. This challenge can be thought of as a missing data or observability problem, which can ultimately bias the analysis and interpretation of EHR-based health outcomes research [17,18]. External pharmacy dispensing databases present a potential solution to bridge the information gap regarding prescription fills [19-21], thereby improving our ability to identify patients who have received a specific medication [22].

While commercial pharmacy databases such as Surescripts have begun to be integrated into EHR databases, there have been few studies that have directly linked Surescripts to EHR data for research purposes. Such linkages can be challenging due to the differences in how these various data streams are accessed and updated, and the different ontologies used for medications across systems. In this study, we present a process to link Surescripts and EHR data. To evaluate the utility of this linkage and the impact of evaluating prescriptions versus pharmacy fill data, we conducted an analysis of prescription fills among children and adolescents who were prescribed psychotropic medication. Identification of psychotropic medication receipt is of particular importance in pediatric populations [23-26]. The management of these conditions requires medication adherence and regular follow-up with a provider to ensure that the patient is being adequately treated and that the side effects of these medications are minimal and acceptable to the patient and their family. Notably, access to these medications can vary significantly based on social drivers of health, patient and family attitudes, insurance, and national and global supply chains [26,27]. Given the need for continued evaluation once a psychoactive medication is prescribed, we specifically evaluated patient and prescriber characteristics and the association between a pharmacy fill for a psychoactive medication and subsequent follow-up with the prescribing provider.

## Methods

### Study Environment and Data

This study included retrospective data from pediatric patients less than 18 years of age who received new psychotropic medication prescriptions within the Duke University Health System (DUHS). DUHS is a medium-sized health system in Durham, North Carolina. DUHS contains over 100 pediatric-serving clinics and is the primary health care provider for approximately 86% of children living in Durham county [28]. All EHR data were extracted from the Duke Clinical Research Datamart, an extract of structured DUHS EHR data that are curated using the standards of the PCORnet common data model [29]. The datamart additionally includes Surescripts prescription fill data, which are reconciled with patient charts within the Epic System. Surescripts facilitates the electronic transmission of prescriptions from EHR to pharmacies and also receives prescription dispense data from most major US pharmacies and pharmacy benefit managers; as of 2020, 96.5% of pharmacies in North Carolina are Surescripts-enabled [30,31]. Data are provided daily based on pharmacy reports and integrated into the EHR system for patient record matching. Pharmacy data for a given patient are updated within the DUHS EHR only upon scheduling of a clinical encounter within the health system.

### Study Population

We identified children younger than 18 years of age with new psychotropic medication prescriptions written at an outpatient visit in 2021. Throughout this paper, the term “prescriptions” refers to psychotropic medication prescriptions. All prescriptions were identified using RxNorm concept unique identifiers (RxCUIs; Table S1 in Multimedia Appendix 1). RxNorm is a normalized naming system, and RxCUIs serve as an identifier assigned to an individual drug entity in RxNorm. A new prescription was defined as a prescription made to a patient who did not have a record of a psychotropic medication prescription or pharmacy fill in the 12 months prior to the date of the index prescription. Because Surescripts data are only incorporated for patients with a subsequent health care encounter, the patient cohort was limited to patients who had any DUHS encounter in 2022.

### Medication Identification and Matching

A primary challenge in working with Surescripts data is the lack of a direct linkage between EHR prescriptions and pharmacy dispensing records. Moreover, the EHR and Surescripts data use 2 different ontologies for medications: RxCUIs and National Drug Codes (NDCs). NDCs provide package-level information about specific drugs, while RxCUIs can correspond to multiple NDCs for similar drug products with the same active ingredient, strength, and dose form but different package sizes or manufacturers. We first mapped the RxCUIs from the EHR to NDCs in the Surescripts data, incorporating the drug name to ensure a correct match between RxCUIs and NDCs [32,33]. Second, using data from 2021 through 2022, we matched prescriptions with dispensing records by identifying the nearest fill date that occurred after the prescription date. Thus, prescriptions with matching NDCs and a fill closest to the prescribing date were considered potential matches. A cutoff of days was ultimately applied to define a valid prescription fill, as this interval aligned with an observed inflection point in the Kaplan-Meier curve of filling prescriptions (see Results section).

### Contextual Variables

We assessed differences between patients who did versus did not fill their prescriptions on 22 contextual variables, including demographic information, clinical characteristics, encounter characteristics, and service use history (Table S2 in Multimedia Appendix 1). Variables included age, sex, race or ethnicity, BMI, the Area of Deprivation Index (ADI) state rank [34], diagnosis (one or more) at the prescribing encounter, prescription of a selective serotonin reuptake inhibitor (the predominant medication class for depression and certain anxiety disorders) versus other type of psychotropic medication [35], the presence or absence of a completed Patient Health Questionnaire-9 (PHQ-9), the PHQ-9 score (categorized as 0‐4=nonminimal, 5-9=mild, 10-14=moderate, 15-19=moderately severe, and 20-27=severe for patients who had a PHQ-9 in the 2 weeks prior to the index prescription) [36], the number of psychotropic medication prescriptions at the index encounter, the number of psychotropic medication prescriptions in 2021, insurance type at the index encounter [37], characteristics of the prescribing provider and clinic, including provider specialty, and health system service use history in 2020 or 2021, including outpatient, inpatient, and well-child visits [38].

### Data Analysis

We initially fit a Kaplan-Meier curve to assess the time from the encounter at which the prescription was created to when the prescription was filled. We then described the demographic and clinical characteristics of patients who did versus did not fill their prescriptions. Continuous variables were summarized using median and IQR. Categorical variables were presented as the number (percentage) for each group. The standardized mean difference was used to measure the magnitude of differences between patients who did versus did not fill their prescriptions, with a standardized mean difference greater than 0.10 indicating an imbalance between groups [39].

To further assess systematic differences between patients who filled their prescriptions and those who did not, we developed a predictive model to identify patients who filled their prescriptions. We applied the least absolute shrinkage and selection operator (LASSO) regression [40] to evaluate the predictability of prescription fills. Data were split into training and testing sets in a 9:1 ratio, with a 5-fold cross-validation conducted to optimize the regularization parameter using the training data and applied the final model to the test data. We evaluated the model performance via the area under the receiver operator characteristic curve (AUROC).

Finally, we evaluated subsequent health care use for patients who did versus did not fill their prescriptions. Using data through 2022, we conducted a time-to-event analysis to identify associations between whether a psychotropic medication prescription was filled and the time until a follow-up encounter with the prescribing provider. We limited eligible follow-up encounters to occur within 90 days of the encounter with the prescription. The period was chosen to provide a sufficient window for capturing relevant follow-up care, as this time frame allows patients to schedule appointments around potential barriers, such as availability or transportation issues, and aligns with common clinical practices for monitoring initial medication responses within 6‐8 weeks [41]. We estimated the marginal hazard ratio (HR) using the Cox proportional hazards model. We derived propensity scores (PSs) using predicted probabilities from the LASSO regression model (described in the Data Analysis section). Inverse probability weighting of PSs was used to adjust for potential confounders (ie, the contextual variables described earlier) by balancing the distribution of covariates between groups. This corrected for the bias that might arise from systematic differences between those who filled their prescriptions and those who did not. All analyses were conducted in R (version 4.1.3; R Foundation for Statistical Computing).

### Ethical Considerations

This study was declared exempt by the DUHS institutional review board (PRO00091342). The institutional review board determined that the study posed minimal risk to participants, as it involved retrospective analysis of deidentified EHRs and pharmacy dispensing data. Patient privacy and data confidentiality were maintained by using deidentified datasets, and access to sensitive information was restricted to authorized study personnel.

## Results

**Figure 1.**
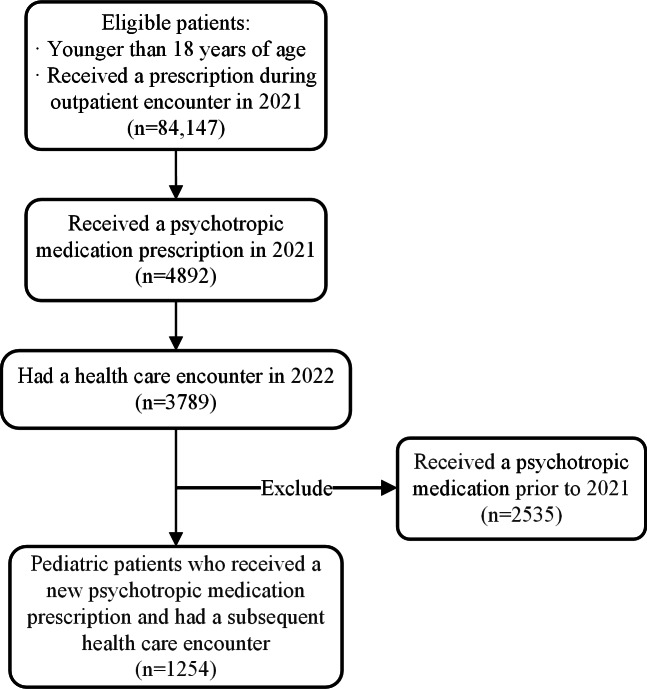
CONSORT (Consolidated Standards of Reporting Trials) diagram.

**Figure 2.**
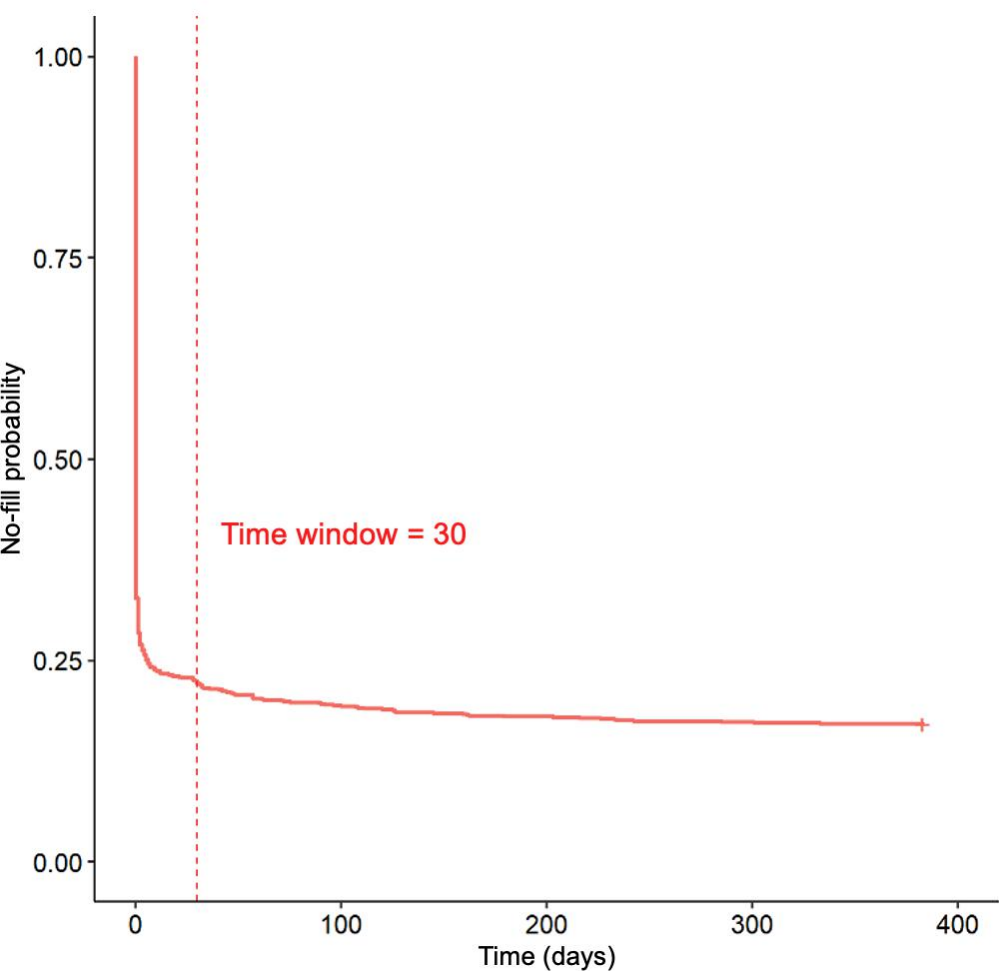
Kaplan-Meier curve depicting time from prescription receipt to prescription fill.

We identified 1254 pediatric patients with new psychotropic medication prescriptions in 2021 (Figure 1). In total, 976 (77.8%) patients had a fill within 30 days of the prescribing encounter, with only 64 prescription fills occurring more than 1 month after the prescribing encounter (Figure 2). Among a total of 1040 patients who filled their prescription, 950 (91.3%) filled it within 7 days, with 844 (81.2%) filling the prescription on the day of the encounter. We then set 30 days as the cutoff for a valid prescription fill. We found that 278 (22.2%) patients did not fill their prescriptions. These patients would have been misclassified as receiving psychotropic medication if only prescriptions were used to identify recipients of these medications.

We evaluated the demographic and clinical characteristics of patients who filled versus did not fill their prescriptions (Table 1). Notably, the 2 groups were similar in terms of race or ethnicity, age, and rates of inpatient and emergency department encounters. We observed a slight difference in sex, with more female patients receiving prescriptions and also a slightly greater proportion of female patients among those who filled their prescriptions. We additionally observed differences in patient BMI, ADI rank, outpatient health care use, and insurance type. Among patients who did not fill their prescriptions, we observed slightly higher BMIs, higher ADI ranks (indicating that patients lived in a more disadvantaged neighborhood), and a greater proportion of patients with public insurance or who were self-pay. Completion of a well-child visit, a marker of health care engagement and receipt of preventive care, was less common among patients who did not fill their prescriptions, but well-child visit rates did not differ significantly in the year after prescription receipt. We additionally observed differences related to the visit type and prescribing provider among patients who did versus did not fill their prescriptions, with a greater proportion of primary care prescribers among patients who ultimately filled their prescriptions. We found that patients diagnosed with anxiety and those who received a prescription for a selective serotonin reuptake inhibitor were more likely to be among those who filled their prescriptions. Patients who had not taken a PHQ-9 recently or who had a PHQ-9 score less than 5 were less likely to fill their prescriptions. Half of the patients (n=635, 50.6%) did not have a recent PHQ-9 score recorded, though some patients may have received the PHQ-9 modified for Adolescents, which was not extracted for this study. There was a slightly higher median number of psychotropic medication prescriptions throughout the year among patients who filled their initial prescription compared to those who did not (median 2, IQR 1-3 vs median 1, IQR 1-2).

**Table 1.** Demographic and clinical characteristics of patients stratified by prescription fill status.

Patient and prescriber characteristics	Overall (N=1254)	Prescription filled (n=976)	Prescription not filled (n=278)	SMD^a^
Age (years), median (IQR)	14 (12-16)	14 (12-16)	14 (12-15)	0.035
**Sex, n (%)**				0.115
Female	838 (66.8)	664 (68)	174 (62.6)	
Male	416 (33.2)	312 (32)	104 (37.4)	
**Race or ethnicity, n (%)**				0.102
Hispanic	137 (10.9)	106 (10.9)	31 (11.2)	
Non-Hispanic Asian	35 (2.8)	25 (2.6)	10 (3.6)	
Non-Hispanic Black	242 (19.3)	195 (20)	47 (16.9)	
Non-Hispanic White	700 (55.8)	544 (55.7)	156 (56.1)	
Other	140 (11.2)	106 (10.9)	34 (12.2)	
BMI, median (IQR)	21.90 (18.89-26.78)	21.62 (18.89-26.10)	22.86 (18.94-28.04)	0.164
ADI^b^ state rank, median (IQR)	3 (2-5)	3 (2-5)	3 (2-6)	0.193
Well-child visit in 2020, n (%)	541 (43.1)	448 (45.9)	93 (33.5)	0.257
Well-child visit in 2021, n (%)	725 (57.8)	571 (58.5)	154 (55.4)	0.063
Outpatient visit in 2020, n (%)	960 (76.6)	764 (78.3)	196 (70.5)	0.179
Inpatient visit in 2020, n (%)	10 (0.8)	6 (0.6)	4 (1.4)	0.082
Emergency department visit 2020, n (%)	55 (4.4)	42 (4.3)	13 (4.7)	0.018
**Primary payer, n (%)**				0.305
Public	558 (44.5)	404 (41.4)	154 (55.4)	
Private	670 (53.4)	554 (56.8)	116 (41.7)	
Self-pay	26 (2.1)	18 (1.8)	8 (2.9)	
**Provider type, n (%)**				0.103
Physician	931 (72.8)	715 (73.3)	216 (77.7)	
Other provider type	323 (25.8)	261 (26.7)	62 (22.3)	
**Prescribing provider specialty, n (%)**				0.384
Primary care	1037 (82.7)	833 (85.3)	204 (73.4)	
Psychiatry	56 (4.5)	47 (4.8)	9 (3.2)	
Other specialty	151 (12)	88 (9)	63 (22.7)	
Unknown specialty	10 (0.8)	8 (0.8)	2 (0.7)	
**Prescribing clinic type, n (%)**				0.655
Primary care	913 (72.8)	760 (77.9)	153 (55)	
Behavioral health	100 (8)	87 (8.9)	13 (4.7)	
Other clinic type	140 (11.2)	82 (8.4)	58 (20.9)	
Unknown	101 (8.1)	47 (4.8)	54 (19.4)	
**Diagnosis at prescribing visit, n (%)**				
Depression	327 (26.1)	262 (26.8)	65 (23.4)	0.080
Anxiety	670 (53.4)	572 (58.6)	98 (35.3)	0.481
ADHD^c^	175 (14)	138 (14.1)	37 (13.3)	0.024
Headache	185 (14.8)	149 (15.3)	36 (12.9)	0.067
Number of prescriptions^d^ at the prescribing encounter, median (IQR)	1 (1-1)	1 (1-1)	1 (1-1)	0.153
Number of prescriptions^d^ in 2021, median (IQR)	1 (1-2)	2 (1-3)	1 (1-2)	0.31
SSRI^e^ received, n (%)	1074 (85.6)	858 (87.9)	216 (77.7)	0.273
**PHQ-9^f^ score severity, n (%)**				0.39
Nonminimal	133 (10.6)	94 (9.6)	39 (14)	
Mild	126 (10)	100 (10.2)	26 (9.4)	
Moderate	132 (10.5)	117 (12)	15 (5.4)	
Moderately severe	125 (10)	108 (11.1)	17 (6.1)	
Severe	103 (8.2)	89 (9.1)	14 (5)	
Not taken	635 (50.6)	468 (48)	167 (60.1)	

a SMD: standardized mean difference.

b ADI: Area Deprivation Index.

c ADHD: attention-deficit/hyperactivity disorder.

d It refers to psychotropic medication prescriptions.

e SSRI: selective serotonin reuptake inhibitor.

f PHQ-9: Patient Health Questionnaire-9.

Using these patient-level variables, we fit a LASSO regression model to discriminate between patients who did versus did not fill their prescriptions. The model achieved an AUROC of 0.816, demonstrating good discriminative ability, and further indicating that clinical and demographic variables differ significantly between patients who did versus did not fill their prescriptions.

**Figure 3.**
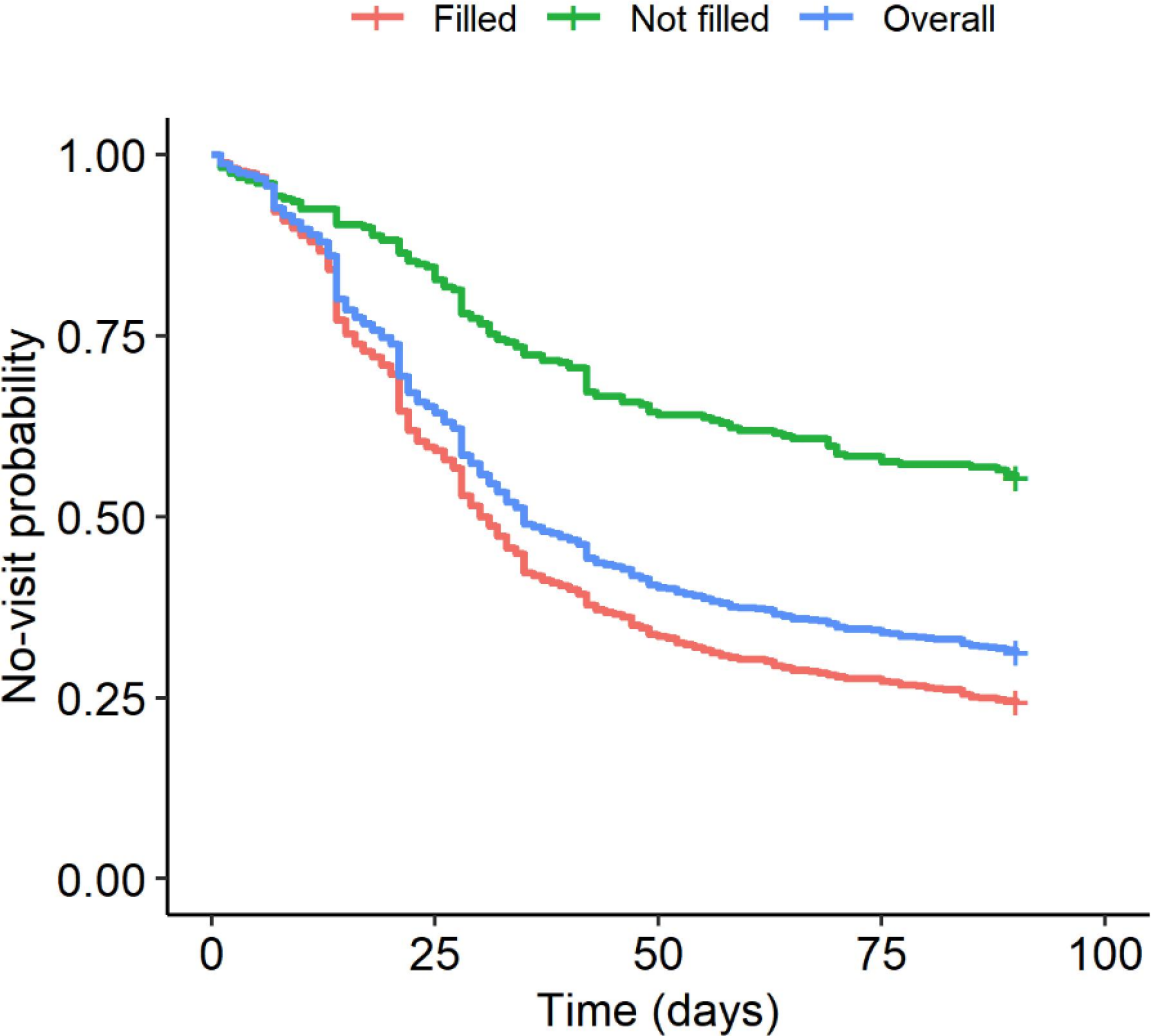
Kaplan-Meier curve depicting days until follow-up visit for patients who did versus did not fill their psychotropic medication prescriptions.

We next evaluated the time to follow-up visit with the prescribing provider between patients who did versus did not fill their psychotropic medication prescriptions (Figure 3). Among patients who filled their prescriptions, 737 (75.5%) had a follow-up visit compared to 123 (44.3%) of those who did not. Patients who filled their prescriptions were more likely to have a subsequent appointment with their prescribing provider than patients who did not fill their prescriptions (marginal HR 1.673, 95% CI 1.463‐1.913). To account for potential systematic differences between these 2 groups, we derived PSs using predicted probabilities from the LASSO regression model. Inverse probability weighting of PSs was used to adjust for potential confounders. After weighting, we calculated the propensity-weighted HR as 1.447 (95% CI 1.257‐1.665).

## Discussion

### Principal Findings

While EHRs contain data on prescriptions, they often lack information on whether these prescriptions are subsequently filled. We sought to evaluate how prescription fill information influenced our understanding of which patients actually receive a prescribed medication and whether a prescription is filled is associated with subsequent health behaviors. We evaluated prescription fill rates among a cohort of pediatric patients who had received prescriptions for psychotropic medications and found that approximately 22.2% (n=278) did not fill their prescriptions. Moreover, there were systematic differences between patients who did versus did not fill their prescriptions. We additionally found that whether a prescription was filled was associated with the likelihood of having a follow-up visit with the prescribing provider. These findings demonstrate that pharmacy dispensing data provide important insights into medication use and contextual information about which populations receive medications and how medication receipt may influence health system interactions and patient outcomes. Importantly, the inclusion of prescription fill data was required to understand how medication receipt may influence subsequent health behavior.

Most prescriptions were filled within 1 week of prescription receipt, with 844 (81.2%) of those who filled doing so on the day of the prescribing encounter. Because these were all new prescriptions, it is likely that patients or their caregivers were motivated to fill the prescriptions to address an acute concern. We found that a greater proportion of female patients filled their psychotropic medication prescriptions compared to male patients. Our finding is consistent with the study by Chua et al [42], which found that female adolescents had a substantially higher monthly antidepressant dispensing rate compared to male adolescents between March 2020 and December 2022. We also found that non-Hispanic White patients and publicly insured patients were more prevalent among the group of patients who did not fill their prescriptions. Children and families with public insurance, such as Medicaid, are more likely to experience adverse social drivers of health [43], which may make it more challenging to follow up with treatment recommendations, including filling a prescription. Our finding that private insurance is associated with higher prescription fill rates is consistent with trends observed in other populations, such as adults on antihypertension medications [44]. We found that a greater proportion of patients filled their prescriptions from a primary care or psychiatry provider compared to those who did not fill. Patients and families may have felt more confident in a course of treatment from a provider with behavioral health expertise than from providers practicing in other specialties. Results indicated that patients with an anxiety disorder were more likely to fill their prescriptions. Prior research has shown that almost three-quarters of pediatric patients who were newly diagnosed with an anxiety disorder received a psychotropic medication order within 5 years of their anxiety diagnosis [45]. Our findings revealed that patients who filled their initial psychotropic prescription had a higher median number of psychotropic prescriptions throughout the year. This suggests that patients who engage with their initial treatment might be more likely to continue with psychotropic medication management. Such findings align with literature emphasizing the importance of initial adherence as a predictor of sustained engagement in mental health care [46]. These findings support previous research that highlights the complex factors contributing to medication nonadherence [47].

The pronounced differences between children and adolescents who did versus did not fill initial psychotropic medication prescriptions underscore the potential risk of differential misclassification. This is particularly noteworthy, as differential misclassification can introduce bias in estimating associations [15,48]. For instance, if we rely only on prescription records, we may misclassify some patients who did not pick up their prescriptions as medication users. By incorporating dispensing records, however, we can more accurately identify actual medication users, as only those who filled their prescriptions would be classified as having received the drug. In our analysis, we found that patients who filled their prescriptions were more likely to have a follow-up visit than those who did not. This demonstrates how including dispensing records changes our understanding of patient behavior, providing a clearer picture of which patients are truly receiving the prescribed treatment.

Our LASSO regression model, which achieved an AUROC of 0.816, indicated that patient demographic and clinical factors were useful predictors of whether prescriptions were filled. This signifies the potential for developing a clinical decision support tool to indicate which patients are more likely to fill versus not to fill a prescription. Such a tool could guide providers in either tailoring additional follow-up or adjusting treatment modalities. It is also important to understand the factors underlying the reasons that patients do not fill a prescription, such as social drivers that may present barriers to filling a prescription, lack of education provided about the rationale for the medication and possible side effects, or not feeling comfortable telling a provider that they do not want to start a psychotropic medication. The LASSO model identified characteristics that may influence both prescription filling and follow-up visits. By using these predicted probabilities as PSs in our analyses on the association between filling and follow-up visits, we reduced potential confounding, strengthening the robustness of our findings.

We identified a significant association between filling prescriptions and follow-up visits with the prescribing provider. This result indicates that prescription filling behavior likely influences subsequent health care engagement and also highlights the importance of including prescription fill data in EHR analyses that account for medication receipt. It is recommended that youths who receive a prescription for a psychotropic medication have weekly to biweekly follow-up visits with their prescribing provider for the first 6 to 8 weeks after initiating a psychotropic medication to monitor treatment response and side effects [41]. We found that patients who filled their prescriptions were more likely to have a follow-up visit with their prescribing provider. Conversely, patients who did not fill prescriptions were less likely to have a follow-up visit. While this analysis is not able to establish whether medication adherence drives health care engagement or health care engagement drives medication adherence, this association nonetheless highlights the relationship between adherence to medication and health care interactions. At the very least, a lack of prescription fills could serve as an early marker of a lack of health care engagement. It is possible that patients did not return to care due to access challenges, reluctance to discuss not filling prescriptions with providers, or a perceived lack of necessity for medical monitoring. Future research should aim to understand patient outcomes when recommended treatment is not followed, especially if moderate to severe mental health symptoms are present.

### Strength and Limitations

This study has some limitations. Of the 4892 patients prescribed psychotropic medication in 2021, a total of 1103 (22.5%) were excluded due to the absence of a follow-up appointment in 2022. While this criterion was applied to ensure complete capture of Surescripts data, it may limit the generalizability of our findings to patients with more consistent health care engagement. Additionally, 635 (50.6%) patients lacked a recent PHQ-9 score, and categorizing PHQ-9 modified for Adolescent scores as “not taken” for consistency limits our ability to interpret associations between PHQ-9 scores and prescription fills. Additionally, we focused on prescription filling behavior for a single prescription per patient; consequently, in instances involving multiple prescriptions on different dates, our matching methodology may require refinement to avoid matching the same dispensing record to more than 1 prescription and ensure each medication dispensing record corresponds to a specific prescription. It is challenging to determine whether prescriptions that lacked a corresponding dispensing record were indeed not filled or simply did not align with a dispensation entry. It is also possible that patients filled their prescriptions at non-Surescripts participating pharmacies, such as digital pharmacies or paid for their prescription out of pocket. Nonetheless, the dispensing rates observed align with prior studies [49]. Furthermore, we restricted our analysis to patients who received health care exclusively within the DUHS and focused only on the prescriptions for psychotropic medications rather than examining multiple health systems or a broader range of medication classes. Future work should investigate prescription filling behavior more broadly across different clinical and geographic populations. Finally, our sample size did not allow us to assess differences in clinical outcomes between those who did and did not fill their medications.

### Conclusions

Overall, our study established a process to link EHR prescription data and pharmacy dispensing databases and shows that the inclusion of dispensing data helps identify patients who have received a medication. This approach enhances our understanding of how medication receipt is associated with health care behaviors, such as follow-up visits. Our findings can potentially inform tools to improve patient medication receipt. The inclusion of dispensing data can be used to reduce potential biases in future pharmacoepidemiology studies.

## Supplementary material

10.2196/63740 Multimedia Appendix 1 RxNorm concept unique identifiers for psychotropic medications and descriptions of the contextual variables used to compare patients who did and did not fill their prescriptions.

## Abbreviations

ADI: Area of Deprivation Index
AUROC: area under the receiver operator characteristic curve
DUHS: Duke University Health System
EHR: electronic health record
HR: hazard ratio
LASSO: least absolute shrinkage and selection operator
NDC: National Drug Code
PHQ-9: Patient Health Questionnaire-9
PS: propensity score
RxCUI: RxNorm concept unique identifier
