# Supplementary material for: Linking Electronic Health Record Prescribing Data and Pharmacy Dispensing Records to Identify Patient-Level Factors Associated With Psychotropic Medication Receipt: Retrospective Study
Source: JMIR Med Inform. 2025 Mar 4;13:e63740. doi: 10.2196/63740 (PMC11895725; doi:10.2196/63740)
Supplement: Multimedia Appendix 1 [file medinform-v13-e63740-s001.docx]

**Table S1.** List of RxCUIs for psychotropic medications.

| RxCUI | Drug Name |
| --- | --- |
| 312941 | sertraline 50 MG Oral Tablet |
| 310385 | fluoxetine 20 MG Oral Capsule |
| 312940 | sertraline 25 MG Oral Tablet |
| 310384 | fluoxetine 10 MG Oral Capsule |
| 312938 | sertraline 100 MG Oral Tablet |
| 349332 | escitalopram 10 MG Oral Tablet |
| 856377 | trazodone hydrochloride 50 MG Oral Tablet |
| 351250 | escitalopram 20 MG Oral Tablet |
| 351249 | escitalopram 5 MG Oral Tablet |
| 856783 | amitriptyline hydrochloride 10 MG Oral Tablet |
| 313989 | fluoxetine 40 MG Oral Capsule |
| 402131 | aripiprazole 5 MG Oral Tablet |
| 602964 | aripiprazole 2 MG Oral Tablet |
| 993541 | 24 HR bupropion hydrochloride 150 MG Extended Release Oral Tablet |
| 313990 | fluoxetine 10 MG Oral Tablet |
| 349545 | aripiprazole 10 MG Oral Tablet |
| 993557 | 24 HR bupropion hydrochloride 300 MG Extended Release Oral Tablet |
| 856373 | trazodone hydrochloride 100 MG Oral Tablet |
| 248642 | fluoxetine 20 MG Oral Tablet |
| 311725 | mirtazapine 15 MG Oral Tablet |
| 856834 | amitriptyline hydrochloride 25 MG Oral Tablet |
| 866083 | buspirone hydrochloride 10 MG Oral Tablet |
| 861064 | sertraline 20 MG/ML Oral Solution |
| 476809 | mirtazapine 7.5 MG Oral Tablet |
| 313583 | 24 HR venlafaxine 37.5 MG Extended Release Oral Capsule |
| 313585 | 24 HR venlafaxine 75 MG Extended Release Oral Capsule |
| 1738483 | paroxetine hydrochloride 10 MG Oral Tablet |
| 866094 | buspirone hydrochloride 5 MG Oral Tablet |
| 315223 | sumatriptan 25 MG Oral Tablet |
| 200371 | citalopram 20 MG Oral Tablet |
| 313161 | sumatriptan 50 MG Oral Tablet |
| 313581 | 24 HR venlafaxine 150 MG Extended Release Oral Capsule |
| 283672 | citalopram 10 MG Oral Tablet |
| 349490 | aripiprazole 15 MG Oral Tablet |
| 485496 | aripiprazole 1 MG/ML Oral Solution |
| 351285 | escitalopram 1 MG/ML Oral Solution |
| 866018 | buspirone hydrochloride 15 MG Oral Tablet |
| 856845 | amitriptyline hydrochloride 50 MG Oral Tablet |
| 314111 | mirtazapine 30 MG Oral Tablet |
| 1190110 | fluoxetine 60 MG Oral Tablet |
| 313159 | sumatriptan 5 MG/ACTUAT Nasal Spray |
| 993503 | 12 HR bupropion hydrochloride 100 MG Extended Release Oral Tablet |
| 993691 | bupropion hydrochloride 75 MG Oral Tablet |
| 313160 | sumatriptan 100 MG Oral Tablet |
| 310386 | fluoxetine 4 MG/ML Oral Solution |
| 856364 | trazodone hydrochloride 150 MG Oral Tablet |
| 866111 | buspirone hydrochloride 7.5 MG Oral Tablet |
| 1738495 | paroxetine hydrochloride 20 MG Oral Tablet |
| 903891 | fluvoxamine maleate 50 MG Oral Tablet |
| 197889 | lithium carbonate 300 MG Oral Capsule |
| 993518 | 12 HR bupropion hydrochloride 150 MG Extended Release Oral Tablet |
| 1607617 | 24 HR desvenlafaxine succinate 25 MG Extended Release Oral Tablet |
| 197891 | lithium carbonate 300 MG Extended Release Oral Tablet |
| 856762 | amitriptyline hydrochloride 100 MG Oral Tablet |
| 993687 | bupropion hydrochloride 100 MG Oral Tablet |
| 309314 | citalopram 40 MG Oral Tablet |
| 903887 | fluvoxamine maleate 25 MG Oral Tablet |
| 993536 | 12 HR bupropion hydrochloride 200 MG Extended Release Oral Tablet |
| 197892 | lithium carbonate 450 MG Extended Release Oral Tablet |
| 903884 | fluvoxamine maleate 100 MG Oral Tablet |
| 309313 | citalopram 2 MG/ML Oral Solution |
| 314227 | sumatriptan 20 MG/ACTUAT Nasal Spray |
| 866090 | buspirone hydrochloride 30 MG Oral Tablet |
| 857305 | clomipramine hydrochloride 75 MG Oral Capsule |
| 349553 | aripiprazole 20 MG Oral Tablet |
| 311355 | lithium carbonate 150 MG Oral Capsule |
| 857297 | clomipramine hydrochloride 25 MG Oral Capsule |
| 857301 | clomipramine hydrochloride 50 MG Oral Capsule |
| 808748 | 24 HR venlafaxine 225 MG Extended Release Oral Tablet |
| 349547 | aripiprazole 30 MG Oral Tablet |
| 197893 | lithium carbonate 600 MG Oral Capsule |
| 1738503 | paroxetine hydrochloride 30 MG Oral Tablet |
| 313584 | venlafaxine 37.5 MG Oral Tablet |
| 903873 | 24 HR fluvoxamine maleate 100 MG Extended Release Oral Capsule |
| 313995 | fluoxetine 90 MG Delayed Release Oral Capsule |
| 1738807 | 24 HR paroxetine hydrochloride 37.5 MG Extended Release Oral Tablet |
| 197890 | lithium carbonate 300 MG Oral Tablet |
| 313582 | venlafaxine 25 MG Oral Tablet |
| 283407 | mirtazapine 30 MG Disintegrating Oral Tablet |
| 312242 | paroxetine hydrochloride 2 MG/ML Oral Suspension |
| 313165 | 0.5 ML sumatriptan 12 MG/ML Injection |
| 313586 | venlafaxine 75 MG Oral Tablet |
| 856369 | trazodone hydrochloride 300 MG Oral Tablet |
| 790288 | 24 HR desvenlafaxine 50 MG Extended Release Oral Tablet |
| 727339 | 0.5 ML sumatriptan 12 MG/ML Prefilled Syringe |
| 790264 | 24 HR desvenlafaxine 100 MG Extended Release Oral Tablet |
| 1086778 | vilazodone hydrochloride 20 MG Oral Tablet |
| 314277 | venlafaxine 50 MG Oral Tablet |
| 849450 | naproxen sodium 500 MG / sumatriptan 85 MG Oral Tablet |
| 1086784 | vilazodone hydrochloride 40 MG Oral Tablet |
| 401978 | sumatriptan 10 MG/ACTUAT Nasal Spray |
| 1439810 | vortioxetine 20 MG Oral Tablet |
| 1439812 | vortioxetine 5 MG Oral Tablet |
| 1551468 | 12 HR bupropion hydrochloride 90 MG / naltrexone hydrochloride 8 MG Extended Release Oral Tablet |
| 1657173 | 0.5 ML sumatriptan 8 MG/ML Cartridge |

**Table S2.** Variable descriptions.

| Type | Variable | Definition |
| --- | --- | --- |
| Demographics | Age | Patient’s age calculated at 01/01/2021 |
|  | Sex | Sex at birth; male or female |
|  | Race/ethnicity | Categories: Hispanic, Non-Hispanic Black, Non-Hispanic White, Non-Hispanic Asian, and other race/ethnicity |
|  | BMI | Body mass index (kg/m^2^) |
|  | ADI state rank | ADI is a measure used to determine the relative level of socioeconomic deprivation of a neighborhood based on various indicators of affluence and deprivation including income, education, employment, and housing quality. The state index ranks neighborhoods across North Carolina. ADI state ranks range from 1 to 10, with a score of 1 indicating the least disadvantaged or most affluent neighborhoods, and a score of 10 indicating the most disadvantaged or least affluent neighborhoods. |
| Service Utilization History  Service Utilization History | Well child visit in 2020 | Indication of whether the patient had a well-child visit in 2020 |
|  | Well child visit in 2021 | Indication of whether the patient had a well-child visit in 2021 |
|  | Outpatient visit in 2020 | Indication of whether the patient had an outpatient visit at DUHS in 2020 |
|  | Inpatient visit in 2020 | Indication of whether the patient had an inpatient visit at DUHS in 2020 |
|  | Emergency department visit in 2020 | Indication of whether the patient had an emergency department visit at DUHS in 2020 |
| Encounter | Primary payer | Payment information for the encounter with prescribing  Categories: public, private, self-pay, and other payer types |
|  | Provider type | The professional training of the provider during the prescribing encounter  Categories: physician, other providers, or unknown |
|  | Specialty type | The primary specialty of the provider  Categories: primary care, psychiatry, other specialties, or unknown |
|  | Location type | The location of the encounter  Categories: primary care, behavioral healthcare, other locations, or unknown location |
| Clinical | Diagnosis | Indication of the diagnosis (one or more) at the prescribing encounter |
|  | SSRI medication | Indication of whether the psychotropic medication prescribed to the patient was a selective serotonin reuptake inhibitor (SSRI) |
|  | PHQ-9 score | The score of patient health questionnaire-9 (PHQ-9) taken within 2 weeks before the encounter  Categories: non-minimal (0-4), mild (5-9), moderate (10-14), moderately severe (15-19), severe (20-27), and not taken |
|  | Number of prescriptions at encounter | The number of psychotropic medication prescriptions issued to a patient during their initial prescribing encounter. This count includes all prescriptions provided during the visit, reflecting cases where multiple prescriptions are given at once. |
|  | Number of prescriptions in 2021 | The total number of psychotropic medication prescription received by a patient over the entire year of 2021. This includes all prescriptions filled across multiple encounters and refills throughout the year. |
